# Alcohol and other drug use patterns and services in an integrated refugee settlement in Northern Zambia: a formative research study

**DOI:** 10.1186/s13031-023-00538-5

**Published:** 2023-08-24

**Authors:** Stephanie Haddad, Mbaita Shawa, Jeremy C. Kane, Bertha Bwalya, Megan Sienkiewicz, Grace Kilbane, Veronica Chibemba, Princess Chiluba, Nkumbu Mtongo, Kristina Metz, Mildred Chibwe, Namuchana Mushabati, Allan Zulu, Ravi Paul, Zaliwe Banda, Henry Loongo, Muzi Kamanga, M. Claire Greene

**Affiliations:** 1 Columbia University Mailman School of Public Health, 60 Haven Avenue, New York, NY 10032 USA; 2 Women in Law and Development in Africa, PO Box 31456, Lusaka, Zambia; 3 CARE Zambia, PO Box 36238, Lusaka, Zambia; 4 Johns Hopkins Bloomberg School of Public Health, 615 North Wolfe Street, Baltimore, MD 21205 USA; 5 School of Medicine, University of Zambia, University Teaching Hospital, PO Box 50110, Lusaka, Zambia; 6 Zambia Ministry of Health, PO Box 30205, Lusaka, Zambia

**Keywords:** Refugees, Displaced persons, Zambia, Alcohol use, Drug use, Formative research

## Abstract

**Background:**

Evidence on patterns of alcohol and other drug (AOD) use and how to effectively deliver services to address AOD use in humanitarian settings is limited. This study aimed to qualitatively explore the patterns of AOD use among Congolese refugees in Mantapala Refugee Settlement and members of the surrounding host community and identify potential appropriate intervention and implementation approaches to address AOD use disorders among conflict-affected populations.

**Methods:**

Fifty free listing interviews, 25 key informant interviews, and four focus group discussions were conducted among refugees, host community members, humanitarian implementing agency staff, and refugee incentive workers. These participants were selected based on their knowledge of AOD use and related problems in the settlement and the surrounding host community in northern Zambia.

**Results:**

Cannabis and home-brewed alcohol were the substances that were perceived to be most commonly used and have the greatest impact on the community. Participants reported that self-medication, boredom, and relief of daily stressors associated with lack of housing, safety, and employment were reasons that people used AODs. Participants recommended that programming include components to address the underlying causes of AOD use, such as livelihood activities. Stigma due to the criminalization of and societal ideals and religious beliefs regarding AOD use was identified as a substantial barrier to accessing and seeking treatment.

**Conclusions:**

Our study’s findings indicate the need for services to address AOD use in Mantapala Refugee Settlement. Interventions should consider the social and structural determinants of AOD use.

## Background

In 2022, there were 108.4 million forcibly displaced people globally due to humanitarian emergencies that arose as a result of persecution, human rights violations, conflict, violence, climate change, or events seriously disturbing public order [1]. A humanitarian emergency is an event or series of events that represents a critical threat to the health, safety, security, or wellbeing of a community or other large group of people, usually over a wide area [2]. Of those forcibly displaced, 90.2% were displaced internally or fled to neighboring countries, the majority of which are low- and middle-income countries (LMIC) [1]. Populations impacted by humanitarian emergencies may experience increased vulnerability to alcohol and other drug (AOD) use disorders as a result of experiencing and/or witnessing stress and trauma, including violence, torture, family separation, and loss of homes and livelihoods The prevalence of mental health disorders, such as post-traumatic stress disorder, are high among this population and the comorbidity between mental health and substance use disorder is well documented in the general population [3–5].

A wide range of licit and illicit substances may be used among individuals impacted by humanitarian emergencies, including alcohol, cannabis, sedatives, inhalants, opioids, and stimulants. While there is limited information regarding AOD use among displaced populations, several qualitative and quantitative retrospective and cross-sectional studies suggest that exposure to humanitarian crises and, consequently, potentially traumatic events, migration stress, loss of homes and livelihoods, violence, torture, family separation, and comorbid mental health problems may increase the risk of AOD use and disorder [6–8]. A systematic review of alcohol use among forced migrants found thatfor studies with validated measures, the prevalence of alcohol dependence ranged from < 1-42% and drug dependence ranged from 1 to 20% [7]. The prevalence of alcohol and drug dependence was higher among forced migrants in camp settings compared to community settings [7]. Additionally, qualitative rapid assessments found widespread use of opiates among refugees in Iran and Pakistan. Among this population, opiate use was believed to be linked to a range of health, social and protection problems, including illness, injury (intentional and unintentional), gender-based violence, risky behavior for HIV and other sexually transmitted infections and blood-borne virus transmission, as well as detrimental effects to the household economy [9].

The burden of AOD use and disorder is also estimated to be high incentral and southern Africa, but research on AOD use and disorder among forced migrants within this region is limited [10–13]. In July 2019, the United Nations High Commissioner for Refugees (UNHCR) conducted an assessment of mental health problems among refugees in Mantapala Refugee Settlement in northern Zambia. Among 200 individuals, 18% had an alcohol use disorder and cannabis was frequently used among people who were drinking alcohol [14]. Additionally, humanitarian workers reported that AOD use was prevalent in the settlement and was associated with individual, family, and community consequences [14–17]. Study investigators conducted a site visit in 2019 to discuss the relevance of research and interventions on AOD use and disorder in Mantapala, which corroborated findings from the UNHCR assessment and indicated an interest in exploring patterns of AOD use and potential interventions within this setting [14–17].

As of 2022, 76% of forcibly displaced persons reside in low-resource settings, such as Zambia, where national health systems have limited AOD treatment services [1, 20, 21]. LMICs have a treatment gap for AOD use disorders of 95.7–99%, which has been exacerbated as a result of the COVID-19 pandemic [1, 20–24]. A significant barrier to the improvement of mental health systems in LMICs is funding for mental and AOD use disorders [25]. Additionally, mental health systems in LMICs often do not have specialized treatment for AOD use disorders, and those that do often provide those services stand-alone services as opposed to being integrated with mental and physical health care, which presents additional barriers to access and help-seeking for AOD use disorder [25, 26]. As humanitarian emergencies become increasingly complex and protracted, with the average humanitarian crisis lasting more than nine years [27], refugee settlements have begun to adapt to an integrated approach where refugees and host community members have access to non-discriminatory services, can participate in economic and livelihood opportunities, and share infrastructure to avoid the parallel systems, services, and communities that segregate refugee from host populations [28].

Despite the prevalence of AOD use disorders among individuals impacted by humanitarian emergencies, few receive treatment due, in part, to the limited availability of specialized treatment and prevention services in humanitarian health systems [18, 19]. The limited evidence on patterns of AOD use and how to effectively deliver appropriate and effective services to prevent and treat AOD use in humanitarian settings further contributes to this gap in available AOD services within humanitarian responses. The study aimed to qualitatively: (1) explore patterns of AOD use among Congolese refugees in an integrated refugee settlement and members of the surrounding host community; and (2) identify appropriate intervention and implementation approaches to address unhealthy AOD use among displaced populations and the host community.

## Methods

### Setting

The Democratic Republic of the Congo (DRC) remains one of the most complex and protracted humanitarian crises in the world. Armed conflict resulting in the killing of civilians has resulted in massive and repeated forced displacement of individuals [29]. As of December 2022, 1.1 million individuals have fled to neighboring countries, such as Zambia, to seek asylum as a result of the conflict [30]. This study was conducted in Mantapala Refugee Settlement in Nchelenge District of Zambia which borders the DRC which was established in January 2018, in response to an influx of Congolese refugees in the region due to escalated conflict [32]. Mantapala is an integrated settlement where the refugees coexist and frequently interact with a farming host community. As of 2022, approximately 11,500 refugees were living within Mantapala Refugee Settlement and 7,000 host community members living in 11 surrounding villages [31, 32]. The settlement has basic services including one rural health center, two schools (offering early childhood, primary, and secondary education), one police station, five child-friendly spaces, and a one-stop center for survivors of gender-based violence available to both refugees and host community members [32].

### Participants

Study participants were purposively selected based on their knowledge of AOD use and related problems in the refugee and surrounding community. Participants were eligible if they were 16 + years of age, a member of the refugee or host community residing in the Mantapala area, and had some knowledge related to AOD use, mental health, or related problems in the community. This study included 16–17 year olds because the onset of AOD use often occurs during adolescence and the researchers wanted to ensure that this population was represented. Participant selection was based on guidance and input from local partners, including humanitarian practitioners, health providers, and community leaders. These partners introduced the study to potential participants and asked whether they were interested in being referred to research staff for an interview or focus group discussion (FGD). If interested, a member of the research team obtained informed consent before proceeding with the interview or FGD. Due to limited literacy levels among the participants, verbal informed consent was obtained instead of written informed consent. Verbal consent was obtained and recorded by the research assistant. For participants aged 16 and 17, parental permission and the assent of the potential child subject were obtained prior to participating in an interview or focus group. All participants were fluent in Bemba.

### Procedures

Data were collected by three research assistants trained in qualitative data collection and research ethics between February and March 2022 and involved three phases: (1) free listing interviews (n = 50); (2) key informant interviews (KIIs, n = 25); and (3) focus group discussions (FGDs, n = 4). Free listing interviews are used to rapidly generate and prioritize concepts that fall within a given cognitive domain [33]. In this study, free listing interviews were used to identify the types of alcohol and other drugs that were used in Mantapala and the surrounding host community, understand community perceptions about the relative impact of AOD use on the community, and explore how individuals deal with AOD use problems. Semi-structured KIIs were administered with community members (n = 10), humanitarian implementing agency staff (n = 7), and refugee incentive workers (i.e., refugees who work with organizations and are compensated through a small monetary incentive) (n = 8) to characterize patterns of AOD use, motivations for use, and perceptions of AOD use interventions. Substance use is a sensitive topic and KIIs were used to elicit candid discussions regarding AOD use in the settlement and surrounding host community. FGDs were conducted to explore relevant and acceptable AOD use intervention approaches and to identify a variety of potential program considerations and how different groups of people think or feel about those considerations. Additionally, FGDs were conducted to explore the social dynamics and how people responded to what others in the group were saying (e.g., to explore consensus vs. areas of variable options). Thirty-one individuals participated in four FGDs. Three FGDs had eight participants and the last FGD had seven participants. FGDs were stratified by age and nationality: youth host community members, adult host community members, youth refugees, and adult refugees. All interviews and FGDs were conducted in the Bemba language and transcripts were translated to English for analysis.

### Data analysis

All data analysis was conducted using Microsoft Excel. Free listing data were analyzed to identify commonly used substances among Congolese refugees and Zambian host community members and their perceived degree of impact on the community. The most highly prioritized substance types were identified through the calculation of Smith’s salience index [34], which incorporates the frequency and ranking of substances in the free list in relation to their relative priority and community impact. For each substance we summarized the description provided by participants and, for the different types of alcohol, we estimated the alcohol by volume (ABV) using either what is reported by commercial alcohol producers or, for home-brewed alcohol, through measurements of samples collected by research assistants with permission from community leaders using an alcohol hydrometer.

KIIs and FGDs were analyzed using a thematic approach. Themes in KIIs were identified through inductive coding by one research assistant and reviewed by a second member of the research team. FGDs were coded deductively according to the domains of the Consolidated Framework for Implementation Research (CFIR) by two research assistants [35]. CFIR is a theory-based guide for systematically assessing potential barriers and facilitators to tailor an intervention [35]. CFIR was used to analyze the FGDs as the interview guide elicited information that could be used to contextualize an AOD use treatment intervention among Congolese refugees in the Mantapala settlement and surrounding host community. To achieve inter-coder reliability for the FGDs, two research assistants coded the transcripts individually and then compared their codes. All discrepancies were resolved through discussion with a third member of the research team. All concepts mapped to each CFIR domain (Intervention Characteristics, Outer Setting, Characteristics of Individuals, and Process of Implementation) were then categorized as a facilitator or barrier. Additionally, findings from the FGDs were compared between refugees and host community members; however, the research team was cautious in over-interpreting the differences since there was only one FGD per age and population group. The analyses of the three qualitative data collection methods were presented to the entire research team to confirm that the findings aligned with what was discussed during the KIIs and FGDs.

## Results

### Patterns of AOD use

Four types of alcohol and two non-alcohol substances emerged as the most salient substances used in Mantapala Refugee Settlement and the surrounding host community (Table 1). *Lutuku* was the substance with the greatest perceived impact in the community (Salience = 0.639). Lutuku is a locally distilled watery brew that is usually packaged in drums. One sample collected by our research team was estimated to have an ABV measurement of 48% based on the alcohol hydrometer measurement. Alternative terms for Lutuku included *Kachasu*, *Kanyanga*, and *Kanchina*. The second highest priority substance was *Ibange* which is the local term for cannabis (Salience = 0.326). The remaining substances, which had lower composite salience scores included three types of alcohol (Eagle, a commercially produced beer; *Katata*, a home-brewed alcohol; and *Cinq ‘Cents*, a home-brewed spirit distilled from Lutuku) and Valium. Patterns of AOD use did not differ between the refugees and host community members.

**Table 1:** Priority substances identified in the free listing interviews

Priority Substance Type	Other Names	Type of Substance	Alcohol By Volume (%)	Composite Salience	Substance Description
Lutuku	Kachasu, Kanyanga, Kanchina	Alcohol	48	0.639	Locally distilled watery brew packaged in drums.
Ibange	Cannabis	Cannabis	N/A	0.326	It is green and leafy and takes the shape of Cassava leaves that are smoked.
Eagle		Alcohol	5.5	0.083	Bottled lager beer packaged in brown bottles. Not locally brewed.
Valium	Infinini, Droga, Droug	Anxiolytic, Sedative	N/A	0.069	Tablets. One respondent said that it is often mixed with alcohol
Katata		Alcohol	3.5	0.056	Locally brewed using maize and millet.
Cinq ‘Cents		Alcohol	89	0.049	Distilled alcohol from Lutuku, made from the remains of Lutuku water. One small bottle can get two people drunk according to one respondent.

Key informants noted that both male and female adolescents and adults use these substances. Home-brewed alcohol was identified as a substance of concern as it is often cheaper than commercial alcohol. AODs are often used together because one substance alone is not considered to be strong enough, and this pattern of use is referred to as ‘topping off.”

*“[People in the community] do mix substances. Usually when they are drinking, the drink Lutuku while also smoking Ibange.”* - Community member

Community members also identified that individuals are experiencing withdrawal and dependence symptoms which leads to further AOD use. Indicators of unhealthy AOD use included insulting others, fighting, weight loss, and swollen body parts.

*“The impact of alcohol and drugs on individuals varies according to how their brain is. You find that for some, they use these substances and start causing havoc in the community while others just go home to sleep once they are intoxicated.”* - Community member

When discussing reasons for AOD use in Mantapala and the surrounding community, participants mentioned that AODs are often used out of boredom or lack of entertainment and/or to cope with pressure and stress, reduce life problems and worries, and address past trauma. Participants also mentioned that people with suicidal thoughts turn to alcohol to temper their desire to harm themselves.

*“[If] somebody is depressed they lack interest in doing things so the only thing they can do is to start taking alcohol.”* - Refugee incentive worker

*“Those with mental health issues usually drink just to lessen bad thoughts and find some rest. You never hear them causing disruptions. They drink in moderation.”* - Refugee incentive worker

### Substance-related harms

There was variation in how individuals experienced and perceived AOD-related problems. One key informant noted that unhealthy AOD use did not cause problems for some people while others who have an alcohol use disorder sometimes did not recognize that problems they were experiencing were caused by their drinking. Key informants identified a range of impacts of AOD use on the community, family, and individual. At the community level, participants mentioned violence (including gender-based violence), insults, theft, loss of productivity, loss of respect from the community, destruction of property, and public nudity. Overall, these harms were perceived to contribute to a lack of peace in the community and households. Family-level consequences included negligence of family duties, loss or diversion of household income, disruptions in the learning environment for children, and protection concerns for women and children. At the individual level, unhealthy AOD use was linked to the risk for AOD use disorder, which could lead to negative health outcomes. Humanitarian practitioners noted that AOD use reduces utilization or engagement with healthcare and other services available in the settlement. Community members discussed that AOD use among youth impacts educational attainment, increases the risk of dropping out of school, and negatively affects relationships with their parents and community.

Findings indicate a substantial amount of stigma associated with AOD use. There were instances described of people living with an AOD use problem who were rejected by their family and community, including being barred from religious buildings. Community members mentioned that this stigma arises from the criminalization of AOD use, societal ideals, and religious beliefs.

*“I personally feel scared to be found with people who are found with drugs because they are criminalized. Some are even barred from attending church. There are people in the community that are always at the receiving end of ridicule. Whatever unpleasant thing is tied to their drug use by other community members.”* - Community member

Despite these observations, there were mixed perceptions of the overall importance of addressing AOD use in Mantapala. KII and FGD participants recognized that implementing an AOD treatment program is novel and important to reduce violence and improve livelihoods, mental health, childcare, and nutrition.

*“Once you address [alcohol and other drug use] that means that the root cause of some of the health problems in Mantapala reduce. Because some of these problems or health problems are coming from the abuse, because they lack food, they are not productive and once the issue is addressed then you find that their health will also improve.”* - Humanitarian practitioner

However, others mentioned that they do not think that addressing AOD use was as important as other issues in the settlement, such as malaria.

### Existing services to address AOD use and AOD use disorder

Community members, humanitarian practitioners, and incentive workers all mentioned that there are limited AOD treatment and prevention services available in the settlement. The use of religious practices, police, traditional healers, and existing services offered through the protection sector were existing resources perceived to be relevant for people with AOD use-related problems. Religious practices (i.e., praying, going to church) are also considered a form of treatment for problematic AOD use by community members and incentive workers. Additionally, community members noted that the police are often involved in addressing AOD use problems in the community. When asked how the police respond to individuals who are under the influence of AODs, a refugee community worker said:

*“We usually just counsel them. If the behaviour is too bad, we usually involve the police so that they can at least spend some nights in a police cell as a way of cautioning them. We also threaten them to say if they cause harm or threaten to cause harm to their wives, we will send them to Nchelenge police where they will be incarcerated.”* - Refugee community worker

Several key informants mentioned that individuals with unhealthy AOD use sought support from traditional healers who use herbs mixed into a porridge to induce vomiting to treat their symptoms. Lastly, community members and incentive workers identified that individuals with AOD use disorders are also often referred to the “one-stop center” that provides services related to social protection and gender-based violence to receive short-term counseling.

### Considerations for introducing AOD treatment interventions into integrated refugee and host community settings

FGD participants identified potential barriers and facilitators to implementing AOD interventions in Mantapala that were coded within the following CFIR domains: intervention characteristics, individual characteristics, process, and outer setting. Very few codes were categorized as inner setting; therefore, it is not included in the analysis. Outer setting determinants related to the existing socioeconomic context. FGD participants emphasized the importance of integrating strategies to address daily stressors (e.g., shelter, safety, livelihoods) within AOD interventions, to overcome barriers to help seeking (e.g., stigma, shame, fear, language barriers, lack of awareness), and to navigate the complex environmental and structural realities such as long distances to intervention sites, inadequate transportation, and the lack of specialized services for AOD use. One humanitarian practitioner noted the importance of considering the social dynamics between refugee and host communities and ensuring equitable access to AOD interventions across these populations given that refugees sometimes received (or are perceived to receive) more services than the host community.

“*There is no difference [in access to healthcare] actually. Even our Zambians complain that most of the time you are giving care to these people who have just come instead of giving us, this is our place and we have offered to keep them here but it’s like your put more attention to them than Zambians*” - Humanitarian practitioner

Characteristics of the individuals and organizations implementing AOD use interventions were considered a major determinant of the acceptability of these interventions. To be successful, interventions must build on community strengths and partner with trusted community leaders and reputable organizations throughout implementation. Desirable characteristics of intervention implementers included respect for participants, their time, and confidentiality; patience; strong communication skills; and knowledge about the treatment of AOD-related problems. An anticipated barrier was the preference to seek care from traditional healers and witchdoctors, who were prohibited in the settlement.

*“Our friends from Congo most of them they don’t believe in treatment from the clinic, they prefer someone goes to buy an injection with the medicine and gives a traditional doctor to inject the person.”* - Humanitarian practitioner

Some participants expressed concerns about implementers being from the same communities as the individuals receiving treatment as they feared this could increase the risk of breaching confidentiality. Another concern was that the implementers could be at risk of harm when working with people who could be under the influence of AODs. To mitigate these risks, participants suggested implementing the intervention in areas within the settlement that are easily accessible and not isolated.

FGD participants identified several aspects of the AOD intervention itself and the process of implementation that are essential for its successful implementation. First, when asked what should be prioritized to better address unhealthy AOD use, participants frequently mentioned the importance of community education and sensitization around AOD-related harms as well as the integration of livelihood and recreational activities. Community sensitization was also described as an imperative step in the implementation process for providing education on AOD use, being transparent about the intervention (i.e., its purpose, expectations, and benefits), and promoting the visibility of the organization and intervention implementers. Multiple participants suggested providing incentives to maintain participant and community engagement such as transportation, materials (e.g., backpacks and notepads), and monetary payments. Some participants mentioned the potential benefits of including community members with a history of AOD use disorder in the recruitment and implementation process; however, the FGDs did not reach a consensus as some participants voiced opposition to this suggestion. When asked to discuss program factors that may impact people from joining an AOD use treatment program, a youth host community member stated,

“*People usually follow the steps of upstanding members of society so, if you are going to recruit counsellors with track record of abusing alcohol and other drugs then they will not follow them but they will only follow someone who is known to be an upstanding member of society or one who is known to have reformed from abusing alcohol and other drugs.*” - youth host community member

Youth community members believed that including individuals with a history of AOD use may influence how receptive the community is to the intervention and impact recruitment and retention in the program. However, FGD participants collectively agreed that working in close collaboration with community and humanitarian stakeholders (e.g., Mantapala health clinic staff) was essential to the successful implementation of an AOD intervention.

## Discussion

In this qualitative study, we described the types and patterns of AOD use and related consequences at the individual, family, and community levels in Mantapala Refugee Settlement and the surrounding host community. Cannabis and potent home-brewed alcohol were the most salient substances that were perceived to be most commonly used and have the greatest impact on the settlement and surrounding host community. The consumption of unregulated home-brewed alcohol can present health concerns associated with toxins and adulterants. Furthermore, methanol can be produced during the fermentation process and its consumption in home-brewed alcohol can lead to blindness and death [36]. Consistent with prior research on AOD use among displaced populations and humanitarian settings, the reasons for AOD use included self-medication, boredom, and relief of daily stressors associated with lack of housing, safety, and employment [8, 37–39].

Our findings suggest that little or no AOD use treatment and related services are available in Mantapala Refugee Settlement. This lack of specialized AOD use services in humanitarian settings is consistent with the literature [12, 13, 18]. Some participants expressed the need for more accessible and available AOD use services, while others identified other services and community needs that may be higher priority.

AOD use was heavily stigmatized in Mantapala Refugee Settlement and surrounding host community. Study participants noted that the police are often tasked with addressing AOD use issues in the settlement and surrounding host community. The criminalization of AOD use is one factor that may have contributed to the stigmatization of AOD use. Existing literature emphasizes how the use of the police often exacerbates stigma and discrimination of AOD use and impacts already marginalized or vulnerable populations [40, 41]. Other factors that contributed to the stigmatization of AOD use were societal norms and religious beliefs surrounding AOD use. This is consistent with the literature that has found that stigma regarding AOD use reflects public norms and values [42]. Additionally, some participants used stigmatizing language during their interviews. Language sustains stigma associated with AOD use and can prevent individuals from seeking care. It is important to note that, stigma as a result of language, public norms and values, and the criminalization of substance use is not unique to humanitarian settings, but what is distinct is that the stigma of substance use can be compounded with the stigma of being a refugee or displaced person [43, 44].

Within Mantapala, recommendations for addressing AOD use involved increasing access to services, while also considering the determinants of AOD-related problems. Incorporating livelihood and recreational programming into AOD use services was a recurring suggestion. In a humanitarian context, this would require the health sector to work with economic development programs. These services are often siloed making it difficult to coordinate efforts [45]. Interdisciplinary coordination between humanitarian aid sectors is necessary to effectively implement AOD-use treatment programs in humanitarian settings. Additionally, implementing organizations should invest in building rapport with the community. Trust must be earned for participants to feel comfortable discussing personal and sensitive information, including AOD use. The importance of establishing rapport to improve program implementation is consistent with the literature [46–48]. Fostering trust within the community can be facilitated through partnerships with community organizations and leaders, which may also improve intervention sustainability and capacity building. In a humanitarian setting, trust is an important factor in launching timely and effective emergency responses as many conflict-affected populations have experienced persecution, human rights violations, conflict, and violence.

The lack of differences in AOD use patterns and reasons for use between refugee and host communities identifies an opportunity to strengthen services for both populations. Integrating treatment into existing healthcare systems and making services available to the entire community has the potential to improve health outcomes and reduce stigma and social tensions.

These findings have several implications for research and practice. The most salient substances being used in these communities were cannabis and home-brewed alcohol. Available and commonly used self-report measures may not accurately capture the consumption of these substances and research must focus on developing measurements to accurately and reliably quantify AOD use. Improved measurement is essential for advancing evaluations of the implementation and effectiveness of AOD interventions in refugee and host communities. Our findings suggest that AOD use interventions should consider the underlying causes of AOD use such as livelihood opportunities, basic needs, and protection, while also addressing some of the barriers to engaging with AOD use interventions, such as stigma. This study uncovers the need for better operational guidance on how to integrate AOD use services into humanitarian settings that considers the suggestions identified by participants such as the involvement of community leaders and traditional healers in AOD use services. Additionally, selecting program implementers who are respected and trusted by the community is essential when designing AOD use-specific interventions.

This study possesses limitations and strengths that should be considered when interpreting the results. First, FGDs were not stratified by gender. Research assistants observed that group dynamics and norms may have overrepresented the voices of male participants relative to female participants. Second, with the support of local partners, we sampled people who had knowledge related to AOD use within the study setting. We did not specifically sample individuals with a personal history of AOD use and disorder and did not ask participants to disclose this information. It is possible that the opinions of people with lived experience with AOD use and disorder are underrepresented in this study. Third, study findings may not be generalizable to other humanitarian or non-humanitarian contexts. As for strengths, the use of multiple data collection methodologies and the recruitment of diverse stakeholder groups allowed us to triangulate and corroborate our findings. Furthermore, the research, data collection, and analysis were conducted through interdisciplinary collaboration with humanitarian and academic organizations. Partnering with local organizations across each phase of this research ensured that the methods were culturally appropriate and the findings were contextualized.

## Conclusion

Unhealthy AOD use is a critical public health issue among refugee and host communities, but there are limited services. Our study indicates the need for tailored services to address the unique AOD use patterns and types and that interventions should include elements that address the social and structural determinants of AOD use. Programming should address the underlying causes of AOD use such as psychosocial and mental health, exposure to potentially traumatic events, and difficulties to meet basic needs. While these findings are not unique to addressing substance use in humanitarian settings, these settings have distinct implementation barriers and challenges such as siloed aid services and the double stigma of AOD use being displaced that must be addressed. These findings have several implications for research and practice. Refugee and host communities must be included in AOD use treatment research and more disaggregated data is needed to understand differences in AOD use trends and patterns, service utilization, and treatment outcomes. Additionally, programming to reduce unhealthy AOD use should address the drivers of AOD use and be designed to ensure the security and confidentiality of both the implementers and participants and integrate local community leaders and practices such as traditional healers. Furthermore, AOD use services and treatment should be accessible to both refugee and host communities to ensure that both populations receive equitable care.

## Data Availability

The data used and/or analyzed during the current study are available from the corresponding author on reasonable request.

## List of abbreviations

ABV: Alcohol by volume
AOD: Alcohol and other drug
FGD: Focus group discussion
IRB: Institutional review board
KII: Key informant interview
